# Antioxidant Amelioration of Riboflavin Transporter Deficiency in Motoneurons Derived from Patient-Specific Induced Pluripotent Stem Cells

**DOI:** 10.3390/ijms21197402

**Published:** 2020-10-07

**Authors:** Chiara Marioli, Valentina Magliocca, Stefania Petrini, Alessia Niceforo, Rossella Borghi, Sara Petrillo, Piergiorgio La Rosa, Fiorella Colasuonno, Tiziana Persichini, Fiorella Piemonte, Keith Massey, Marco Tartaglia, Sandra Moreno, Enrico Bertini, Claudia Compagnucci

**Affiliations:** 1 Genetics and Rare Diseases Research Division, IRCCS Ospedale Pediatrico Bambino Gesù, 00146 Rome, Italy; chiara.marioli@opbg.net (C.M.); fiorella.colasuonno@uniroma3.it (F.C.); marco.tartaglia@opbg.net (M.T.); 2 Department of Science, University Roma Tre, 00146 Rome, Italy; valentina.magliocca@opbg.net (V.M.); tiziana.persichini@uniroma3.it (T.P.); 3 Unit of Neuromuscular and Neurodegenerative Diseases, Laboratory of Molecular Medicine, Department of Neuroscience, IRCCS Ospedale Pediatrico Bambino Gesù, 00146 Rome, Italy; alessianiceforo@hotmail.it (A.N.); rossella.borghi@opbg.net (R.B.); sara.petrillo@opbg.net (S.P.); fiorella.piemonte@opbg.net (F.P.); enricosilvio.bertini@opbg.net (E.B.); 4 Confocal Microscopy Core Facility, Research Laboratories, IRCCS Ospedale Pediatrico Bambino Gesù, 00146 Rome, Italy; stefania.petrini@opbg.net; 5 Department of Science, LIME, University Roma Tre, 00146 Rome, Italy; sandra.moreno@uniroma3.it; 6 Department of Psychology, Division of Neuroscience, Sapienza University of Rome, 00185 Rome, Italy; piergiorgio.larosa@hotmail.it; 7 Science Director, Cure RTD Foundation, 6228 Northaven Rd., Dallas, TX 75230, USA; keith.massey@curertd.org

**Keywords:** neurodegenerative disease, motoneurons, mitochondria, oxidative stress, RTD syndrome, riboflavin transporters, antioxidants, iPSCs

## Abstract

Mitochondrial dysfunction is a key element in the pathogenesis of neurodegenerative disorders, such as riboflavin transporter deficiency (RTD). This is a rare, childhood-onset disease characterized by motoneuron degeneration and caused by mutations in *SLC52A2* and *SLC52A3*, encoding riboflavin (RF) transporters (RFVT2 and RFVT3, respectively), resulting in muscle weakness, ponto-bulbar paralysis and sensorineural deafness. Based on previous findings, which document the contribution of oxidative stress in RTD pathogenesis, we tested possible beneficial effects of several antioxidants (Vitamin C, Idebenone, Coenzyme Q_10_ and EPI-743, either alone or in combination with RF) on the morphology and function of neurons derived from induced pluripotent stem cells (iPSCs) from two RTD patients. To identify possible improvement of the neuronal morphotype, neurite length was measured by confocal microscopy after β-III tubulin immunofluorescent staining. Neuronal function was evaluated by determining superoxide anion generation by MitoSOX assay and intracellular calcium (Ca^2+^) levels, using the Fluo-4 probe. Among the antioxidants tested, EPI-743 restored the redox status, improved neurite length and ameliorated intracellular calcium influx into RTD motoneurons. In conclusion, we suggest that antioxidant supplementation may have a role in RTD treatment.

## 1. Introduction

Neurodegenerative diseases (ND), constitute a spectrum of chronic debilitating disorders characterized by irreversible progressive loss of neurons. Although the brain regions and cell types affected in various neurodegenerative disorders are disease-specific, common factors contribute to their pathogenesis, among which progressive destabilization of microtubules, axonal and dendritic degeneration, energy dysmetabolism and oxidative stress are shared events [1,2]. Nearly all NDs share mitochondrial dysfunction, and many are associated with mutations affecting mitochondrial homeostasis [3,4,5,6]. Mitochondria are energy transducing organelles that also play central roles in many other cellular functions, including apoptosis, cell cycle regulation, calcium homeostasis and heme synthesis [1,7]. During oxidative phosphorylation, leakage of electrons from the electron transport chain generates of reactive oxygen species (ROS), such as hydroxyl radical, superoxide anion and hydrogen peroxide [8]. In turn, these species act as signalling molecules, inducing biological responses such as proliferation, migration, and differentiation [9,10], but their overproduction can damage proteins, lipids and nucleic acids, thus impairing normal metabolism [1,9]. Neurons are highly differentiated cells, requiring large amounts of ATP to perform their many complex biological functions. Because neurons have reduced glycolytic capacity, ATP production is highly dependent on mitochondrial bioenergetics, thus enhancing the adverse consequences of oxidative damage in neuronal mitochondria. Of note, the levels of protective antioxidants in the nervous system are lower than those in other tissues, which further make neurons vulnerable cells [10,11].

Because of the increased susceptibility of neurons to redox imbalance, and the role played by oxidative stress in the onset and progression of neurodegenerative disorders, molecules with antioxidant properties are receiving increasing attention for the treatment or prevention of these pathologies [11]. In addition to it being a major component of the mitochondrial bioenergetic system, Coenzyme Q_10_ (CoQ_10_, or ubiquinone) can act as a powerful lipophilic antioxidant outside mitochondria. Supplementation of CoQ_10_ in short-term studies has proved beneficial to patients with Parkinson’s disease [12,13], Huntington’s disease [14,15] and Friedreich’s ataxia [16], among other neurological disorders. Idebenone (IDEB) is a synthetic, less lipophilic analogue of CoQ_10_ used in various diseases associated with respiratory chain dysfunction, including Leber’s hereditary optic neuropathy [17], Leigh syndrome [18], Friedreich’s ataxia [19,20], Alzheimer’s disease and Huntington’s disease [21,22,23]. Compared to CoQ_10_, IDEB crosses the blood-brain barrier more effectively and acts as a free radical scavenger to protect mitochondrial membranes and other, less lipophilic, membranes from lipid peroxidation [24,25,26,27]. In addition to its well-known role as a major natural antioxidant, vitamin C, or ascorbic acid (AA), participates in various enzymatic reactions, including catecholamine synthesis [28], collagen production, regulation of HIF-1α and modulation of glutamatergic transmission [29,30]. EPI-743 is a synthetic, vitamin EB-like para-benzoquinone antioxidant that readily crosses the blood brain barrier, where it can act to increase the biosynthesis of glutathione (GSH), a major natural ROS-scavenging molecule [31,32,33]. EPI-743 has proved effective in short-term treatment protocols for Leigh’s syndrome [34], mitochondrial disease-related epilepsy [35] and Friedreich’s ataxia [36] but, in theory, it could be used in a variety of disorders in which mitochondrial function is impaired. Riboflavin (RF, or vitamin B_2_) is a precursor of flavin adenine dinucleotide (FAD) and flavin mononucleotide (FMN), which are cofactors for many redox enzymes in oxidative phosphorylation and numerous other metabolic pathways. RF is absorbed in the small intestine by three transporters, RFVT1, RFVT2 and RFVT3. In 2010, mutations *SLC52A2* (encoding RFVT2) and *SLC52A3* (encoding RFVT3) were shown to cause the neurodegenerative disorder Brown-Vialetto-Van Laere syndrome (BVVL) which, therefore, designated it as a riboflavin transporter deficiency (RTD) [37]. Collectively, RTDs are rare, autosomal, recessive neurological diseases whose clinical features include ponto-bulbar palsy, limb and axial muscle weakness, sensorineural hearing loss, optic atrophy, ataxia and respiratory compromise [38]. A disorder with a similar presentation, but without deafness, is Fazio-Londe syndrome, which is caused by mutations in *SLC52A3* and is now included in the RTD disease spectrum. The age of onset of RTDs varies from childhood to the third decade, and current therapeutic options are limited. Although supplementation with high-dose RF can be an effective treatment, particularly if started soon after the onset of symptoms, RF supplementation cannot be considered a generally applicable therapy because some RTD patients have responded poorly [39,40,41].

iPSC (induced pluripotent stem cell) technology has allowed the creation of disease models, drug screening and personalized treatments for many genetic disorders [42,43,44,45,46]. The special advantage of using iPSCs is creating patient-specific cellular models by differentiating iPSCs into the cell types affected in the disease, in this case, RTD-motoneurons (RTD-MNs) [47]. Since a murine model accurately recapitulating the human pathology is lacking, the RTD model of iPSC-derived motoneurons (MNs) is of particular interest. The iPSC model allows in vitro reproduction of the molecular mechanisms responsible for progression of RTDs, and study of morphological and functional changes in patients’ cells. A recent study from our group documented altered cell-cell contacts, abnormal mitochondrial ultrastructural features, redox imbalance, abnormal expression of antioxidant enzymes and peroxisomal downregulation using the RTD iPSC model [48] One additional piece of evidence that RTD iPSCs can be used as an informative in vitro model is that, in a different study [49], we reported on cytoskeletal/morphological and functional abnormalities of RTD iPSC-derived MNs which were reverted by combined riboflavin/NAC treatment.

In the present work, we assessed the possible beneficial effects of an array of treatments based on antioxidant molecules, used alone or in combination with RF, against RTD pathology. As an in vitro model for RTD neurological disease, we used MNs derived from fibroblasts obtained from RTD patients reprogrammed into iPSCs. This experimental model was used to assess the rescue of the RTD phenotype by antioxidants and RF, focusing on selected MN differentiation features. These included neurite length measurement utilizing β-III tubulin immunofluorescence, redox status analysis by MitoSOX assay, and calcium imaging after ionomycin administration.

## 2. Results

### 2.1. Antioxidant Treatment Restores Redox Status of RTD MNs

**Figure 1:**
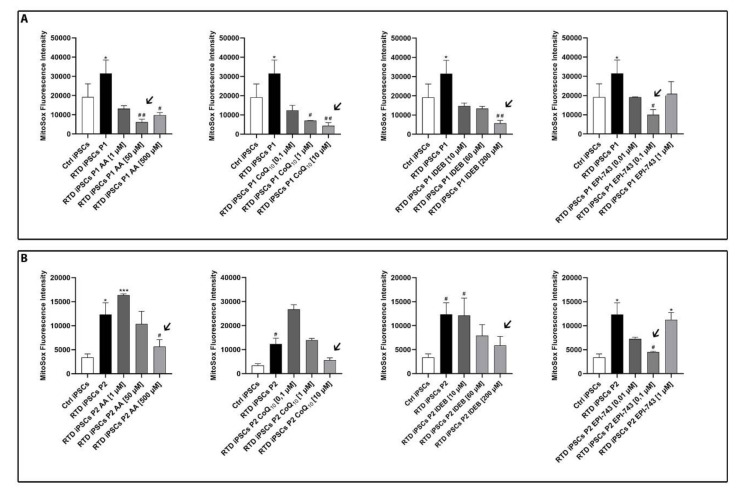
Quantification of superoxide anion in riboflavin transporter deficiency (RTD) induced pluripotent stem cells (iPSCs) following treatment with antioxidants ascorbic acid (AA), CoQ_10_, Idebenone, and EPI-743, showing their effect on RTD iPSCs superoxide anion production. For each RTD cell line, P1 (**A**) and P2 (**B**), the arrows indicate the antioxidant concentration most effective in lowering the levels of superoxide anion. Experiments were conducted in triplicate and values expressed as mean ± standard error of the mean (SEM). According to Kruskal-Wallis tests * *p* < 0.05, *** *p* < 0.001, compared with controls’ group (Ctrl); # *p* < 0.05, ## *p* < 0.01 respect to untreated patients.

To evaluate the RTD cell redox status before and after antioxidant treatments, we performed a MitoSOX Red assay for selective detection of mitochondrial superoxide anions (O_2_^−^) in iPSCs. MitoSOX fluorescence intensity was measured under basal conditions and after antioxidant supplementation to determine the optimal concentration for reduction of O_2_^−^ in RTD iPSCs. Of note, we determined the same optimal concentration for both RTD lines for riboflavin, coenzyme Q_10_, idebenone and EPI-743, whereas for AA, two different optimal concentrations were found and used for the two RTD iPSC lines (Figure 1).

### 2.2. EPI-743 Treatment Is Able to Reduce the Levels of Oxidized Lipids in RTD iPSCs

**Figure 2:**
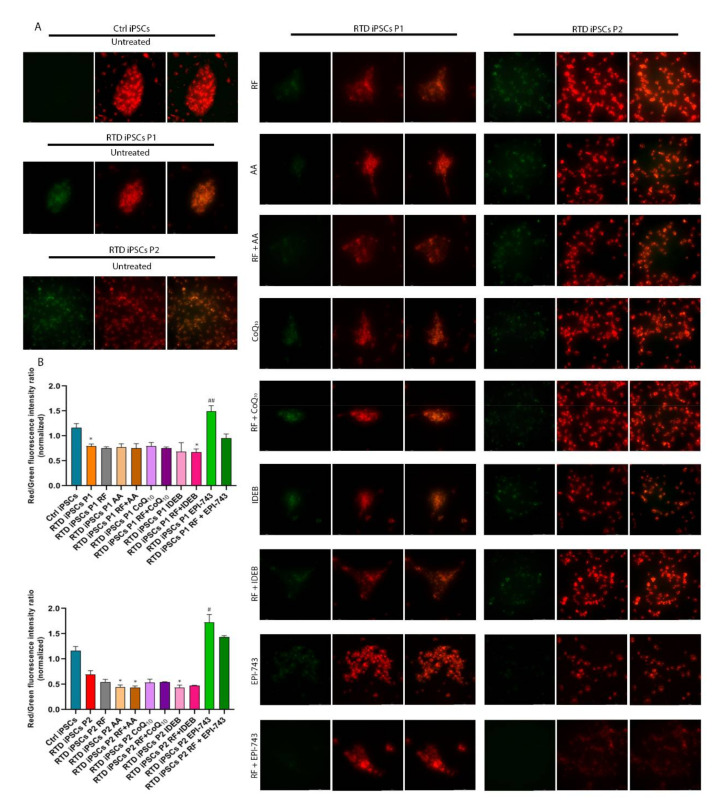
RTD iPSCs show increased lipid peroxidation. (**A**) Fluorescence micrographs of iPSCs labeled with BODIPY using the green (on the left, indicating oxidation of the butadienyl portion of the dye) and the red (on the middle) filter and then overlay of the red (nonoxidized) and green (oxidized) images (in the right column). Colocalization of oxidized and reduced BODIPY fluorescence appears in yellow. Bar = 100 μm. Control and RTD iPSCs were incubated for 45 min with BODIPY 581/591 C11. Treatment with EPI-743, but not other antioxidants, results in significantly reduced levels of oxidized lipids as shown by the shift of green to red fluorescent signal. (**B**) Bar graph reporting the quantitative analyses of the BODIPY experiments performed on control and RTD iPSCs. Values are expressed as mean ± SEM. According to Kruskal-Wallis test * *p* < 0.05 compared with controls iPSCs; # *p* < 0.05, ## *p* < 0.01, respect to untreated patients.

To determine if oxidative stress increased lipid peroxidation in RTD iPSCs, we used the oxidation sensitive lipid sensor, BODIPY 581/591 C11. This probe emits red fluorescence in reduced conditions, shifting to green fluorescence when the lipid portion of the dye is oxidized [48]. Incubating RTD iPSCs with BODIPY showed increased levels of green fluorescence, indicating increased levels of oxidized lipids in RTD iPSCs compared to control cells. We used this endophenotype of iPSCs to assess the antioxidant properties of EPI-743 as it is reported to have 15-lipoxygenase as a direct target (see discussion for details). Importantly, the supplementation of RF, AA, CoQ10, idebenone (IDEB) and combined antioxidant + RF was not able to reduce the levels of oxidized lipids (as shown by the shift of green to red fluorescent signal, Figure 2A,B). Interestingly, the measured level of oxidized lipids was lower following EPI-743 administration (Figure 2A,B).

### 2.3. Morphological Analyses Show that EPI-743 Ameliorates the RTD Phenotype

**Figure 3:**
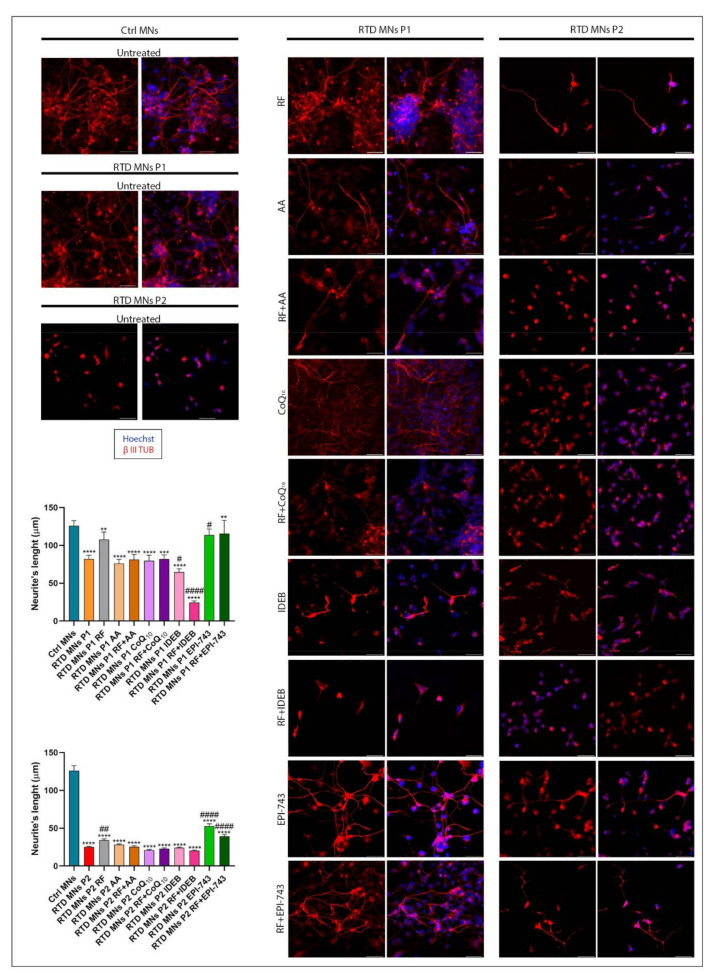
Analysis of neurite length following antioxidant treatment of MNs derived from RTD patient-derived iPSCs. Immunofluorescence images of β III tubulin (in red) show shorter neurites in RTD MNs compared to control cells. In both P1 and P2 RTD MNs, treatment with AA, CoQ10 and IDEB fails to cause significant changes. RF and EPI-743 ± RF causes improvement in neurite length for RTD MNs. Nuclei are counterstained with Hoechst (in blue). Scale bars = 50 μm. Data derived from four independent experiments, and values are expressed as mean ± SEM. According to Kruskal-Wallis test ** *p* < 0.01, *** *p* < 0.001, **** *p* < 0.0001, compared with control group (Ctrl); # *p* < 0.05, ## *p* < 0.01, ### *p* < 0.001, #### *p* < 0.0001 with respect to untreated RTD patient MNs.

iPSCs from patients with RTD have been shown in vitro to have abnormal morphology [49]. During in vitro differentiation, the growth and length of neurites can be used as parameters of neuronal maturity [50]. To investigate the possible benefits of antioxidant treatment on RTD MNs, we differentiated control (Ctrl) and RTD iPSCs into MNs using an established protocol [51]. Immunofluorescence localization of the neuronal marker, β-III tubulin (β-III-TUB), was used to delineate neurites and measure their lengths [52]. The results confirmed that RTD MNs have shorter neurites than Ctrl MNs, and that RF treatment increased neurite length, although to a limited degree (as reported in Niceforo et al., [49] submitted). Notably, treatment with EPI-743, but not with AA, IDEB and CoQ_10_, caused almost full recovery of neurite length for P1, considerably improving the morphology of P1′s MNs (Figure 3). Combined administration of RF and antioxidants, however, did not further increase mean neurite length and, for IDEB, the addition of RF substantially reduced neurite length.

### 2.4. EPI-743 Has a Beneficial Effect on the Intracellular Calcium Levels in RTD MNs

**Figure 4:**
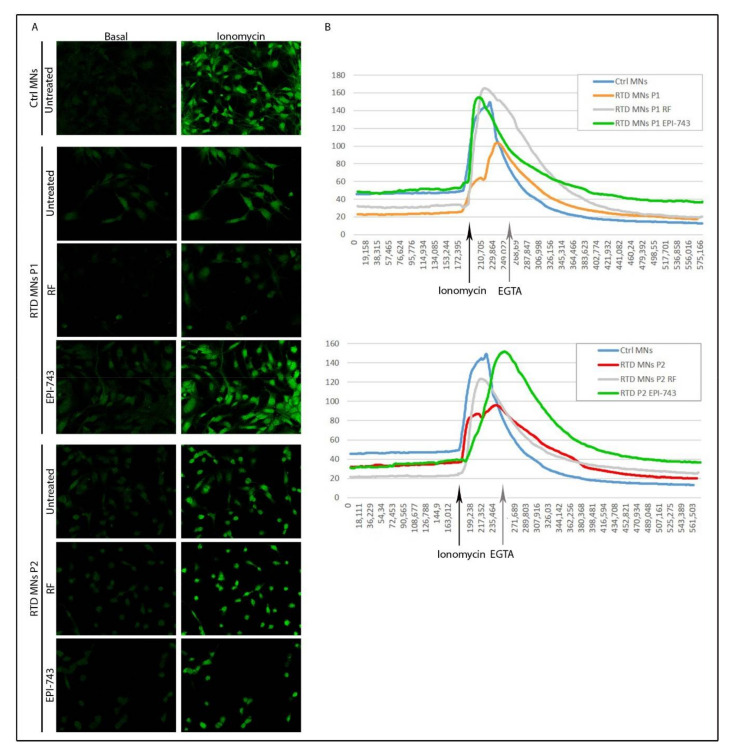
Intracellular Ca^2+^ flux in RTD motoneurons following EPI-743 treatment. (**A**) Confocal images showing changes in intracellular calcium flux in RTD and control MNs before (basal level) and after stimulation with 5 μM ionomycin. (**B**) Graphical representation of the mean fluorescence intensity over time of Control and RTD MNs following RF or EPI-743 treatment, showing an increase in intracellular Ca^2+^ following ionomycin supplementation (indicated by the black arrow) and decrease following addition of EGTA to the medium (30 s following ionomycin in all samples, as indicated by the grey arrow). Experiments were conducted in triplicate.

Intracellular calcium levels play a fundamental role in synaptic activity and in many other biological functions. Consequently, calcium imaging is used to quantify neuronal activity. It is known that control MNs functionally responded to calcium mobilization following ionomycin stimulation, and patient-derived MNs presented altered calcium homeostasis [49]. We confirmed that before ionomycin stimulation (basal conditions), the intracellular calcium levels were lower in both RTD MNs, and that even following ionomycin the maximal peak of intracellular calcium in RTD MNs was decreased compared to Ctrl MNs (Figure 4). Importantly, treatment with RF or EPI-743 considerably, increased intracellular calcium levels. However, despite RFs increasing the maximal intensity of RTD MNs to levels comparable to Ctrl MNs, the basal levels of calcium did not change following RF supplementation. The basal calcium levels of P1 RTD MNs, however, were restored to Ctrl levels following EPI-743 treatment without RF. These results support the notion that EPI-743 improves the intracellular calcium flow in RTD MNs compared to untreated MNs, and that this effect is stronger compared to that observed with RF treatment.

## 3. Discussion

Human iPSC technology has opened the way to research in personalized medicine. Use of iPSCs in in vitro models of rare genetic diseases, such as RTD, makes it possible to design clinically effective therapeutic strategies. Taking advantage of this experimental approach, we created a model of RTD, which proved useful for reproducing cellular and molecular aspects of RTD pathology [48,49,50]. The aim of the present work was to test a novel therapeutic approach for RTD directed to counterbalance ROS overproduction, which represents a major cellular endophenotype associated with RF deficiency. Focusing on morphological alterations and calcium imaging in neurons differentiated from RTD patient-derived iPSCs, we tested the ability of RF in combination with different antioxidants to ameliorate the RTD-associated neuronal phenotype. Of note, antioxidants are currently being used in clinical treatment protocols for mitochondrial and neurodegenerative disorders [11] and in the treatment of individual RTD patients (K. Massey, unpublished observations).

In the present study, we used iPSCs derived from two RTD patients with biallelic mutations in *SLC52A2* and induced them to differentiate in the cell type mostly affected by the disease, the motor neuron. In a recent study by our group, we performed a detailed ultrastructural analysis of mitochondria from RTD patient-specific iPSCs demonstrating dramatic morphological alterations. Specifically, we found several damaged mitochondria with disrupted cristae in RTD iPSCs with an average size significantly larger than in controls. We also performed JC-1 staining to assess the mitochondrial membrane potential of RTD iPSCs showing significantly abnormal membrane potential of RTD mitochondria, as compared to controls [48]. Because RTD cells have increased levels of superoxide anions [48], which can be partially reduced by N-acetyl cysteine (NAC) in combination with RF [49], we investigated the effects exerted by other antioxidants, namely, CoQ_10_, AA, IDEB and EPI-743, on the RTD phenotype. In particular, we explored the effect of combined administration of RF with each of the chosen antioxidants on the morphology and intracellular calcium influx of RTD MNs.

To determine the efficacy of antioxidant molecules in restoring the redox status of RTD cells, we evaluated mitochondrial O_2_^−^ concentration, by performing MitoSOX assays, using different antioxidant concentrations. Our study confirmed that RTD iPSCs have increased levels of O_2_^−^ [48]. The data also showed a general decrease of ROS levels with antioxidant treatment and what was the most effective concentration for each of these compounds, and they indicated the most effective dosage for each of these compounds on iPSCs as they were administered to the cell culture media during neuronal differentiation from iPSCs to neuronal progenitors and neurons. The RTD MNs were then treated with the selected concentration of each antioxidant, and the morphofunctional changes during MN differentiation measured. Among the tested treatments, only RF and EPI-743 successfully restored normal neuronal morphology and neurite length. More specifically, for P1, nearly normal values of this important maturity parameter were reached, whereas for P2, which displayed more severe morphological abnormalities in the basal state, the abnormal shortening of neurite length was only partially ameliorated. The other antioxidants, administered alone or in combination with RF, were documented to be unable to fully restore the RTD neuronal morphotype to normal. We previously observed that NAC and RF + NAC have positive effects on RTD neuronal phenotype [49] but, in the present work, the other antioxidants (IDEB, AA, CoQ_10_) did not show similar behavior except for EPI-743 (Figure 3). Further investigation will be necessary to understand why RF+NAC ameliorated the endophenotype of RTD neurons with respect to RF alone (as reported in Niceforo et al., [49] submitted), while RF + antioxidants (IDEB, AA, CoQ_10_) did not show similar positive results. 

Some patients are currently treated with a combination of RF and antioxidants with some apparent success. One possible explanation for the lack of RTD phenotype amelioration following RF + antioxidant treatment in this work (particularly considering the severe effects obtained with RF + IDEB) may be related to the fact that neuronal cultures were treated during differentiation without the metabolic support provided by glial cells. Therefore, treating cocultures of astrocytes and neurons with RF + antioxidants might restore the normal neuronal phenotype. We nevertheless hypothesize that RF + antioxidants have positive effects on the RTD neuronal phenotype, and with this aim we plan to further investigate the biological mechanisms underlying RTD pathogenesis and to test the effect of different antioxidant species and concentrations on neuronal morphology and function. In fact, it is possible that the concentration of antioxidants tested on iPSCs using the MitoSOX, used to determine the best effective concentrations, is not optimal for iPSCs differentiating into neurons and/or on neurons. In addition to this, we would like to point out that all antioxidants decreased the superoxide anion content in the iPSCs, but not the lipid peroxidation. In fact, only treatment with EPI-743 was active in reducing the levels of oxidized lipids (Figure 2)

As a functional feature of neuronal cells used to test the efficacy of RF and EPI-743, intracellular calcium levels were considered. Changes in Ca^2+^ concentration play a critical role in neuronal development, apoptosis, synaptic plasticity and signal transduction. Mitochondrial function and Ca^2+^ signalling are intimately linked because Ca^2+^ regulates mitochondrial energy homeostasis. Indeed, studies have identified anomalies in Ca^2+^ homeostasis in many, if not all, neurodegenerative diseases [53,54]. To study the functional properties of RTD MNs, we carried out calcium imaging analyses, demonstrating overall amelioration of RTD pathological features following EPI-743 supplementation. RTD MNs of P1 treated with EPI-743 reached basal and peak levels of intracellular Ca^2+^ comparable to those of Ctrl MNs, while RTD MNs of P2 treated with EPI-743 showed an increased peak of intracellular calcium influx, even though basal levels remained considerably below those of control MNs. Consistent with the morphological studies, the other antioxidant molecules failed to significantly ameliorate altered calcium influx in RTD cells.

**Figure 5:**
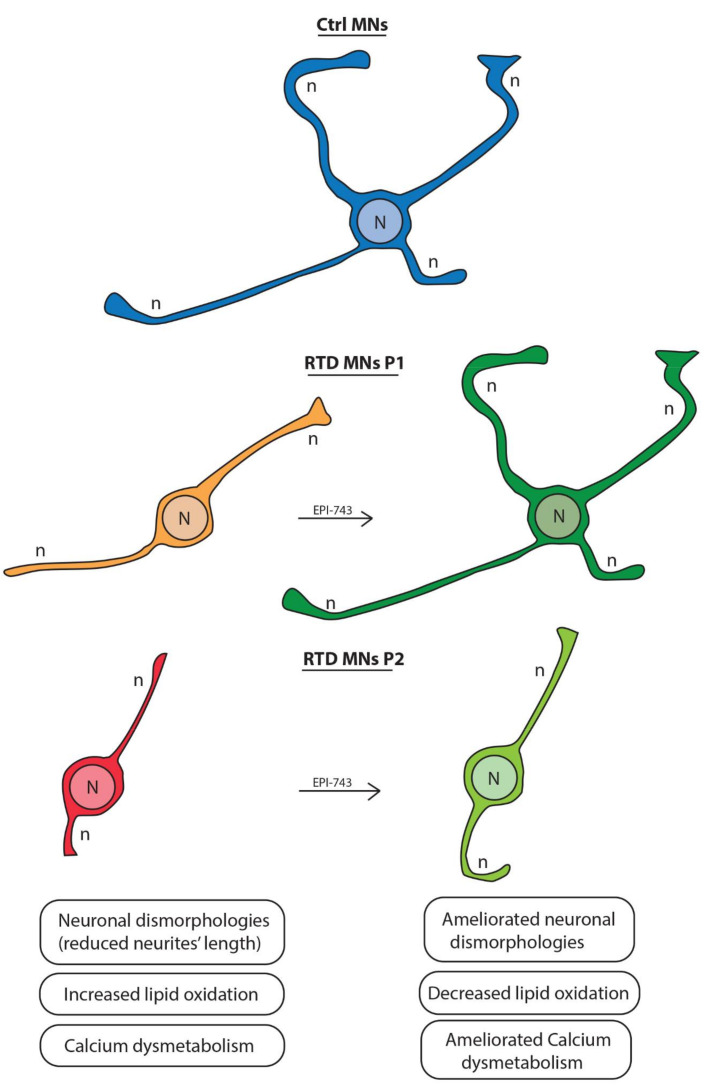
Schemae showing the morphological changes of the RTD MNs before and following treatment with EPI-743. The drawing depicts neurons with short neurites in RTD P1 and P2, but following EPI-743 treatment they extend longer neurites that, for RTD P2, are very similar to Ctrl MNs, while, for RTD P1 neurite length is improved but still not comparable to that of Ctrl MNs. N = Nucleus. n = neurite.

The reason why only EPI-743 was successful in reducing the morphofunctional abnormalities of RTD MNs remains to be determined, but some speculations are possible (Figure 5). For example, EPI-743 has recently been shown to have beneficial effects in some neurodegenerative disorders, such as Huntington’s disease, Friedreich’s ataxia, Leigh’s syndrome and Leber’s hereditary optic neuropathy, by complex and concerted mechanisms [55,56]. EPI-743 also prevented ferroptosis in vitro by specifically inhibiting the activity of 15-lipoxygenase [35,57], which is responsible for inflammatory mediator biosynthesis [58]. In addition, EPI-743 increased the expression of nuclear factor erythroid 2-related factor 2 (Nrf2), an important regulator of cellular resistance to ROS damage [59,60,61]. Nrf2 controls the basal and induced expression of an array of pathways for regulating the physiological and pathophysiological outcomes of oxidant exposure. The Nrf2 signaling pathway shows many levels of regulation, and different redox-active drugs may promote differential patterns of Nrf2 induction, as recently described in Petrillo et al. 2019 [60]. Besides redox homeostasis, Nrf2 modulates pluripotent stem cells through the regulation of pluripotency factors, metabolism and cellular stress responses [62]. Moreover, it has been demonstrated that some stem cell models are associated with an increased level of Nrf2 [63,64]. In particular, Nrf2 expression is high in human embryonic stem cells [65], and Nrf2 inhibition by Keap1 overexpression alters metabolic reprogramming and reduces the efficiency of iPSCs colony formation [66]. Therefore, Nrf2 activation is highly controversial and its role in our in vitro cell model of RTD syndrome remains to be clarified. EPI-743 is likely to act in a more complex fashion, compared to other antioxidant molecules, because it interferes with multiple cell pathways that culminate in neuronal dysfunction. This would account for a pleiotropic beneficial effect of EPI-743 in restoring a normal neuronal phenotype. However, we can also speculate that the efficacy of EPI-743 treatment of RTD cells is linked, in part, to its ability to act on 15-lipoxygenase. For example, we observed that the switch between red and green fluorescence of BODIPY-staining of lipid droplets [67,68] revealed an increase in oxidized lipids in RTD cells compared to control iPSCs and, that following EPI-743 treatment, the levels of oxidized lipids were reduced. Since EPI-743 is a fat-soluble compound with a favourable preclinical profile, further evaluation of EPI.743 as a possible adjunct for the treatment of RTDs may be warranted. In particular, we would like to clarify that BODIPY is used as marker of lipid peroxidation (because it renders it susceptible to lipid peroxidation), in particular that deriving from the formation of autoxidation chain-carrying lipid peroxyl radicals [69]. Importantly, the MitoSOX probe differs from BODIPY because it selectively recognizes the mitochondrial superoxide anion. Indeed, once in the mitochondria, MitoSOX reagent is oxidized by superoxide and exhibits red fluorescence; then the reagent is readily oxidized by superoxide, but not by other ROS- or reactive nitrogen species (RNS)–generating systems, and oxidation of the probe is prevented by superoxide dismutase. By contrast, the BODIPY probe reacts with oxy, peroxy or hydroxyl radicals, but not with superoxide, nitric oxide, transition metals or peroxides per se [70].

This study provides evidence that, among the antioxidants tested, EPI-743 is able to significantly ameliorate the morphological and functional alterations detected in RTD MNs.

## 4. Materials and Methods

### 4.1. Derivation of iPSCs:

The studies involving human samples were conducted in compliance with the Code of Ethics of the World Medical Association (Declaration of Helsinki) and with national legislation and institutional guidelines (Local institutional ethical committee of Ospedale Pediatrico Bambino Gesù, Ref 1702_OPBG_2018, date of approval 11 February 2019). Human fibroblast cell lines were obtained from two RTD patients with informed consent (ethical committee approved at the Ospedale Pediatrico Bambino Gesù). Fibroblasts were generated from skin biopsies following informed consent. Generation of iPSCs was performed by nonviral transduction with episomal technology by SBI System Biosciences (USA). Control iPSCs were derived from fibroblasts of two healthy individuals. Patients’ iPSCs were derived from fibroblasts of two RTD patients with mutations in *SLC52A2*. Patient one (P1) has the mutations c.155C>T and c.935T>C (named RTD P1), and patient two (P2) has the mutations c.155C>T and c.1255G>A (named RTD P2). P1 developed macrocytic anemia and dysphagia at 3 months of age. At one year, optic atrophy, axial muscle weakness, sensory ataxia and respiratory compromise were noted, and at two years bilateral sensorineural hearing loss occurred. Patient P1 is now nine years and has remained neurologically stable since beginning riboflavin (75 mg/kg QID) and antioxidant therapy at 2.5 years of age. At two years P2 developed exercise intolerance with dyspnea and cyanosis together with progressive dysphonia. At three years, P2 developed progressive shoulder and axial muscle weakness and bilateral sensorineural hearing loss and reduced visual acuity [71]. P2 required hospitalization for acute respiratory failure and aspiration pneumonia before his 4th year. He died soon thereafter.

### 4.2. Maintenance of iPSCs

The iPSCs obtained from reprogramming were maintained in culture on 6-well plates coated with Matrigel (Cod. 354277, Corning, New York, NY, USA) in mTeSR1 plus (Cod. 05826, Stem Cell Technologies, Vancouver, Canada and incubated at 37 °C, 5% CO_2_. The medium was changed every other day, and when the cells reached 7080–% confluence they were split and transferred to new plates.

### 4.3. Drug Treatments

Ascorbic acid (AA) was purchased from Sigma Aldrich (Cod. A5960, St. Louis, MI, USA), dissolved in deionized water (stock solution of 200 mM) and cells treated for 24 h with a final concentration of 50 μM on RTD P1 iPSCs and 500 μM on RTD P2 iPSCs. CoQ_10_ was purchased from Sigma Aldrich (Cod. C9538), dissolved in chloroform (stock solution of 30 mM) and cells treated for 24 h with a final concentration of 10 μM. Idebenone (IDEB) was purchased from Sigma Aldrich (Cod. I5659), dissolved in deionized water (stock solution of 30 mM) and cells treated for 24 h with a final concentration of 200 μM. EPI-743 was purchased from BioElectron Technology Corporation (Mountain View, CA, USA), dissolved in DMSO (Dimethyl sulfoxide, stock solution of 1 mM) and cells treated for 24 h at a final concentration of 0.1 μM. The antioxidant compounds (CoQ_10_, AA, IDEB) were administered during neuronal differentiation at every medium change. EPI-743 was administered once a week during four weeks of neuronal differentiation.

### 4.4. BODIPY Staining

Cells were incubated with 5 µM BODIPY 581/591 C11 (D3861, Thermofisher Scientific, Waltham, MA, USA) for 45 min, using a modified protocol from [68]. Cells were viewed with a Leica DMi8 fluorescence microscope (Leica Microsystems, Germany) and raw images were analyzed with Image J to obtain quantitative data (the red/green fluorescence intensity was used).

### 4.5. Differentiation of iPSCs into Motor Neurons

The iPSCs were differentiated into MNs by adapting the protocol proposed by Corti et al. [51]. Cells were kept in culture for 10 days with the NeuroCult NS-A Basal Medium Human medium (Cod. 05750, Stem Cell Technologies, Vancouver, Canada), and, on the 10th day 0.1 µΜ of retinoic acid (Cod. R2625, Sigma Aldrich, St. Louis, MI, USA) was added to the medium and it was replaced on alternate days until the 17th day when, in addition to retinoic acid, dorsomorphin 2 µΜ (Cod. P5499, Sigma Aldrich) and activin A 3 ng/mL were added (Cod. 120-14E, PeproTech, Rocky Hill, CT, USA). From day 25 until the end of the differentiation, BrainPhys Neuronal Medium (Cod. 05790, Stem Cell Technologies) was used as medium, and ascorbic acid 200 μM (Cod A4403, Sigma Aldrich), GDNF 2 μg/mL (Cod 450-10, PeproTech), BDNF 10 ng/mL (Cod. 450-02, PeproTech), SM_1_ (Cod. 05711, Stem Cell Technologies) and N_2_ (Cod. 17502-001, Thermofisher Scientific) were added to the medium [51].

### 4.6. MitoSOX Red Assay

The rate of O_2_^−^ production was measured using the mitochondria-specific probe MitoSOX Red Mitochondrial Superoxide Indicator (Cod. M36008, Thermofisher Scientific, Waltham, MA, USA). This fluorogenic dye permeates living cells and quickly and selectively enters mitochondria, where it is oxidized and measures the increase in fluorescence intensity (emission: ~ 510/580 nm). When the cultured cells reached 80 to 90% confluence, they were preliminarily treated with RF alone or + antioxidants, and the next day they were harvested and centrifuged at 1200 rpm for 5 min, washed in PBS and incubated with MitoSOX Red Probe 5 μM for 45 min at 37 °C. At the end of the incubation time, the fluorescence intensity was measured by the EnSpire Multimode Plate Reader (Perkin Elmer), and the data analyzed. Data were normalized based on protein amount, as determined by the Bicinchoninic acid assay (Pierce BCA Protein Assay Kit, ThermoFisher Scientific).

### 4.7. Immunofluorescence

The differentiated cells were fixed with 4% paraformaldehyde for 10 min at room temperature (RT) and treated with a permeabilizing and blocking solution containing 1× phosphate-buffered saline (PBS), 5% bovine serum albumin (Vector Laboratories, Burlingame, CA, USA) and 0.1% Triton X-100 (Sigma). Incubation was performed with the primary antibody against β III-tubulin (Cod T2200, Sigma Aldrich) diluted 1: 500 and maintained at RT for 2 h. The secondary antibody conjugated with AlexaFluor 555 (Thermofisher Scientific) was diluted 1: 500 and incubated at RT for 1 h. The nuclei were counterstained using Hoechst 33342 (Cod. H3570, Thermofisher Scientific).

### 4.8. Morphometric Analysis

For all images, we used a Leica confocal microscope, and measurements of the length of the neurites were made using the Leica LAS-AF software (associated with a Leica confocal microscope). The neurite length was performed following [50].

### 4.9. Confocal Microscopy

Confocal optical sectioning was performed with a Leica TCS-SP8X (Leica Microsystems, Germany) equipped with a White Light Laser (WLL) source and a 405 nm diode laser. Samples were photographed at 20× to perform quantitative evaluation of neurites’ length. Representative images were assembled using Adobe Photoshop CS6 software (Adobe Systems Inc., USA).

### 4.10. Calcium Imaging

In this work we followed the methodology proposed by Glaser et al. (2016) [72]. The differentiated cells were grown on 35 mm optical plates (Cod. 81156, Ibidi, Gräfelfing, Germany), coated with Matrigel, and washed with HBSS (Cod. 14025-050, Gibco, Carlsbad, CA, USA). The solution containing the Fluo-4 probe (Cod. F10489, ThermoFisher Scientific) was added and, after 15 min incubation, recording begun. After 3 min, 5 μM of ionomycin (Cod. I24222, Invitrogen, Waltham, MA, USA) was added to the cells to record the maximum fluorescence peak. The addition of 30 mM EGTA followed (Cod. SLBR7504V, Sigma Aldrich). Recording ended after 10 min. Live recording was conducted with a frame rate of 2 fs/sec, magnification 20× with a 1024 × 800 format and an electronic zoom at 2.0 using the SP8X Leica confocal microscope equipped with a resonant scanner for fast imaging (8.0 MHz), and a stage incubator (OkoLab, Italy) allowing maintenance at 37 °C and a humidified atmosphere with 5% CO_2_. For each biological replicate, 10 to 15 cells were measured. The changes in fluorescence intensity over time were represented in the curve graph reported.

### 4.11. Statistical Analyses

Data were represented using mean and standard error of the mean (mean ± SEM) where the distribution was normal. Multiple technical replicates and biological replicates were utilized for all experiments and a minimum of three independent experiments were performed for each assay. Significance was tested using Student’s *t* test or ANOVA (parametric tests) for normally-distributed data, and Kruskal-Wallis (nonparametric tests) when normal distribution could not be assessed. GraphPad-Prism software (Prism 8.0.2, GraphPad Software) was used to analyze the data.

## Abbreviations

AA	Ascorbic acid
AD	Alzheimer disease
ATP	Adenosine triphosphate
BVVL	Brown-Vialetto-Van Laere syndrome
β-III-Tub	β III tubulin
Ca^2+^	Intracellular calcium
COQ_10_	Coenzyme Q_10_
GSH	Glutathione System
HD	Huntington’s disease
IDEB	Idebenone
LHON	Leber hereditary optic neuropathy
MNs	Motoneurons
ND	Neurodegenerative disease
PD	Parkinson’s disease
RF	Riboflavin
RFTV	Riboflavin transporter
ROS	Reactive oxygen species
RTD	Riboflavin transporter deficiency
